# Anticipated help-seeking for cancer symptoms before and after the coronavirus pandemic: results from the Onco-barometer population survey in Spain

**DOI:** 10.1038/s41416-021-01382-1

**Published:** 2021-04-14

**Authors:** Dafina Petrova, Marina Pollán, Miguel Rodriguez-Barranco, Dunia Garrido, Josep M. Borrás, Maria-José Sánchez

**Affiliations:** 1 CIBER of Epidemiology and Public Health (CIBERESP), Madrid, Spain; 2 Escuela Andaluza de Salud Pública (EASP), Granada, Spain; 3 Instituto de Investigación Biosanitaria ibs.GRANADA, Granada, Spain; 4 National Center for Epidemiology, Health Institute Carlos III, Madrid, Spain; 5 University of Granada, Granada, Spain; 6 Department of Clinical Sciences, University of Barcelona, Barcelona, Spain; 7 Bellvitge Biomedical Research Institute, Hospitalet, Spain; 8 NHS Cancer Strategy, Ministry of Health, Hospitalet, Spain; 9 Department of Preventive Medicine and Public Health, University of Granada, Granada, Spain

**Keywords:** Cancer epidemiology, Cancer screening, Signs and symptoms, Public health, Diagnosis

## Abstract

**Background:**

The patient interval—the time patients wait before consulting their physician after noticing cancer symptoms—contributes to diagnostic delays. We compared anticipated help-seeking times for cancer symptoms and perceived barriers to help-seeking before and after the coronavirus pandemic.

**Methods:**

Two waves (pre-Coronavirus: February 2020, *N* = 3269; and post-Coronavirus: August 2020, *N* = 1500) of the Spanish Onco-barometer population survey were compared. The international ABC instrument was administered. Pre–post comparisons were performed using multiple logistic and Poisson regression models.

**Results:**

There was a consistent and significant increase in anticipated times to help-seeking for 12 of 13 cancer symptoms, with the largest increases for breast changes (OR = 1.54, 95% CI 1.22–1–96) and unexplained bleeding (OR = 1.50, 1.26–1.79). Respondents were more likely to report barriers to help-seeking in the post wave, most notably worry about what the doctor may find (OR = 1.58, 1.35–1.84) and worry about wasting the doctor’s time (OR = 1.48, 1.25–1.74). Women and older individuals were the most affected.

**Conclusions:**

Participants reported longer waiting times to help-seeking for cancer symptoms after the pandemic. There is an urgent need for public interventions encouraging people to consult their physicians with symptoms suggestive of cancer and counteracting the main barriers perceived during the pandemic situation.

## Background

Healthcare systems around the world are under unprecedented pressure due to the coronavirus pandemic, which has disrupted prevention and treatment services for non-communicable diseases such as cancer.^1^ The pandemic has led to a significant reduction or complete suspension of cancer screening programs and reassigning of resources from cancer to COVID-19 care.^2–4^ These disruptions will likely lead to significant delays in cancer diagnosis and treatment, which will translate into more cases diagnosed in later stages, with serious implications for patient survival, quality of life, and healthcare costs.^2,5^ In support of this expectation, a notable reduction in the number of cancer diagnoses during the pandemic has already been documented in several countries.^4,6,7^

In European countries, primary care is the gateway to access health services for symptomatic patients. Unfortunately, experts have warned that, due to the exceptional burden suffered by primary care services, delays in responses to suspected cancer symptoms are inevitable.^8,9^ The pandemic is likely to impact negatively all intervals of the diagnostic pathway, including the patient interval—the time elapsed between symptom onset and the first consultation with a physician.^9^

Patients may be less likely to consult for symptoms during the pandemic due to fear of being infected, limited capacity to use telemedicine, or diverse perceived barriers.^2,8^ This delay may be particularly important in patients experiencing more vague cancer symptoms such as weight loss, fatigue or changes in bowel habits^8^ or in younger patients (e.g. <65) who have family and work obligations complicated by the pandemic.

Spain has been one of the countries hit hardest by the coronavirus^10^ and, hence, an increase in delays along the cancer care pathway is expected due to the extreme and continuous pressure exerted on the health system. A nation-wide study reported coronavirus seroprevalence of 5% in May 2020^11^ and on February 25, 2020, more than 3,180,000 cases and 68,800 deaths due to COVID-19 were registered.^12^ A recent study of 37 tertiary centres from all around the country showed that the number of new cancer patients decreased by 21% during the first pandemic wave (March–June 2020) compared to the same period in 2019.^7^

Experts recommended that efforts be made to encourage patients to seek help for potential cancer symptoms during the pandemic by addressing current delays and barriers to help-seeking and identifying groups at high risk of delays.^9,13^ To better understand the changes that might have occurred after the first wave of the pandemic, the aim of the current study was to compare anticipated help-seeking for cancer symptoms and perceived barriers in the Spanish population before and after the pandemic.

## Method

Data were obtained from the *Onco-barometer 2020*, a population-based survey conducted by the Spanish Association against Cancer (www.aecc.es). The *Onco-barometer* assesses knowledge and attitudes towards cancer. In 2020, for the first time, the survey included a module on help-seeking for cancer symptoms. The original data collection protocol was interrupted by the announcement of the pandemic and the state of emergency by the Spanish government (14 March 2020) and was renewed once conditions allowed. This generated two survey waves: pre-Coronavirus (10 February 2020 until 13 March 2020, *N* = 3269 respondents) and post-Coronavirus (24 August 2020 until 08 September 2020, *N* = 1500 respondents), offering us the opportunity to measure population-level changes in anticipated help-seeking.

Computer-assisted interviews were carried out by a specialised research market company under a contract by the Spanish Association against Cancer. A two-stage sampling design was used. First, stratified random sampling proportional to the population sizes of the Spanish Autonomous Regions was used for household selection. Then, sampling units were selected by applying sex and age quotas with one interview per household. The distribution mobiles/landlines was 50%/50%. Men and women, 18 years or older, and able to speak Spanish were eligible.

### Variables

Anticipated times to help-seeking and perceived barriers were measured with the internationally validated Awareness and Beliefs about Cancer (ABC) questionnaire by the International Cancer Benchmarking Partnership (ICBP).^14^ For the current study, it was translated to Spanish and back-translated to English by fluent speakers of both languages. Where applicable, answer options were adjusted to the circumstances of the Spanish health system. The questionnaire was then pilot tested with a small purposive sample of 10 respondents (50% female) aged 55 or older with diverse educational backgrounds. No comprehension problems or other issues arose, so the pilot was interrupted, and no changes were made to the first version.

### Anticipated times to help-seeking

Respondents were asked how long they would wait before consulting their physician for 13 cancer warning signs. Answers were unprompted (respondents answered freely) and were then assigned to one of the categories provided by the ABC instrument.^14^ Besides considering each symptom separately, following previous studies,^15–17^ we categorised the answers to each item as ‘delayed’ or ‘not delayed’ and assigned one point for each ‘delayed’ answer, thereby generating a total delay score for each respondent (0–13). We are not aware of any national or international guidelines defining the maximum recommended time for help-seeking for symptoms compatible with cancer, so the cut-off used to define a ‘delayed’ answer was waiting >1 week, based on the distribution of responses for the majority of symptoms. Alternative cut-offs were also used (see below). Answers indicating that the respondent would contact another healthcare professional (0.0–0.3%) were dropped for this calculation because the delay was not clear.^18^

### Perceived barriers to help-seeking

Respondents were asked whether each of the following reasons would make them delay consulting their physician: being embarrassed, being worried about wasting the doctor’s time, being worried about what the doctor may find, and not having enough time to go to the doctor.^14^ Respondents were also asked what additional barriers would make them delay consulting their physician. Answers to this question were coded into meaningful categories by two independent coders who were blind to the wave each answer belonged to. Disagreements were resolved by discussion.

A global barrier score was then calculated (0–5), whereby each respondent was assigned one point for each reported barrier based on the four predefined questions and the additional barrier reported in the open-ended question.

### Demographic characteristics

Data were collected on sex, age, marital status, socioeconomic position (categorised in seven groups following the methodology of the Spanish National Health Survey and the Spanish Epidemiology Society^19^), personal history of cancer, and having a close family member with cancer.

### Analyses

Chi-square tests were used to compare the two waves on categorical items. To investigate the effect of wave on help-seeking for each symptom and perceived barrier, we computed unadjusted (OR) and adjusted (adjOR) odds ratios based on logistic regressions. Adjusted ORs were based on models including all socio-demographic and cancer history variables as covariates. To identify general patterns and socio-demographic groups potentially more vulnerable in the pandemic situation we used the total delay and barrier scores. In particular, we conducted multiple Poisson regressions, in which we tested for significant interactions of wave with sex and age. Sample weights were applied in all analyses. Sensitivity analyses reported in the supplement investigated differences in help-seeking times using alternative delay scores based on cut-offs of 2 and 3 weeks, respectively, to define delayed responses. In the case of missing data, analyses were based on complete cases.

## Results

Response rate was 64.1% (65.8% in the pre- and 61.0% in the post-wave). Demographic characteristics of respondents in both waves are displayed in Table 1. Compared to the pre-wave (*N* = 3269), respondents in the post-wave (*N* = 1500) were more likely to be male and single.

**Table 1:** Demographic characteristics of respondents and *p*-values from chi-square tests comparing the pre and post waves.

	Wave		*p*-value
	Pre	Post	
	*N* = 3269	*N* = 1500	
Sex			
Male	41.6%	47.6%	<0.001
Female	58.4%	52.4%	
Age			
18–24 years	7.7%	9.7%	0.116
25–34 years	14.0%	13.1%	
35–44 years	19.0%	19.6%	
45–54 years	18.9%	20.1%	
55–64 years	16.1%	15.0%	
≥65 years	24.4%	22.6%	
Socioeconomic position			
GROUP I. Directors and managers of establishments with 10 or more employees and professionals traditionally associated with university degrees	13.0%	11.6%	0.051
GROUP II. Directors and managers of establishments with fewer than 10 employees and professionals traditionally associated with university degrees	17.2%	15.9%	
GROUP III. Intermediate occupations: employees of the administrative type and professionals supporting administrative management	19.2%	21.2%	
GROUP IV. Free-lancers/self-employed	1.5%	1.4%	
GROUP V. Supervisors and workers in qualified technical occupations	8.8%	10.1%	
GROUP VI. Qualified workers of the primary sector and other semi-qualified workers	28.6%	25.6%	
Group VII. Unskilled workers	11.8%	14.2%	
Civil status			
Married or cohabiting	52.5%	47.9%	0.038
Single	31.7%	36.1%	
Separated or divorced	8.1%	8.5%	
Widowed	7.2%	7.2%	
Other	0.4%	0.4%	
Personal cancer history			
No	90.6%	91.1%	0.628
Yes	9.4%	8.9%	
Close family member with cancer			
No	26.3%	24.5%	0.176
Yes	73.7%	75.5%	

### Anticipated help-seeking

**Fig. 1:**
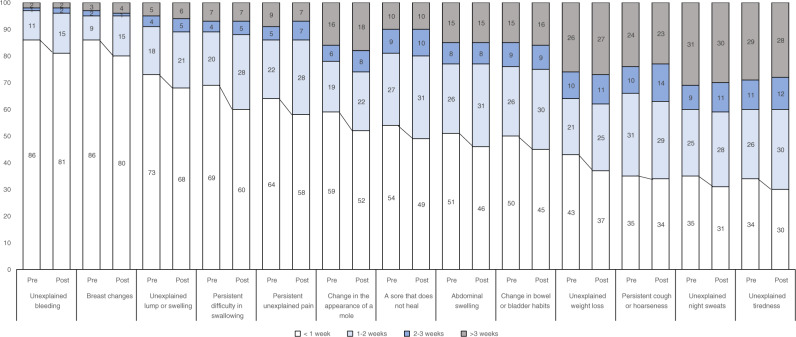
Anticipated help-seeking as a function of wave. Percentage of respondents reporting anticipated help-seeking times within the different time categories for 13 possible cancer warning signs as a function of wave (pre vs. post).

Anticipated help-seeking was generally prompt for most symptoms. Importantly, there were differences in the answer distributions between the pre- and post-waves for virtually all symptoms (Table 2). These differences became apparent when considering the cut-off of 1 week, whereby the percentage of respondents consulting their physician later than a week after symptom onset increased in a consistent and significant manner from the pre- to the post-wave for all cancer symptoms, with the exception of persistent cough or hoarseness (Fig. 1). Overall, the odds of seeking care later than a week after symptom onset increased by about 20–50% in the post wave (Table 3). The amount of change varied by symptom with the largest relative increases observed for breast changes, unexplained bleeding, and persistent difficulty swallowing.

**Table 2:** Responses to the question ‘How long would you wait to consult your physician from the moment you detect the symptom for the first time?’ for 13 possible cancer warning signs as a function of wave (pre vs. post).

	Unexplained bleeding		Breast changes		Unexplained lump or swelling		Persistent difficulty in swallowing	
	Wave		Wave		Wave		Wave	
	Pre	Post	Pre	Post	Pre	Post	Pre	Post
	*N* = 3269	*N* = 1500	*N* = 1913	*N* = 796	*N* = 3269	*N* = 1500	*N* = 3269	*N* = 1500
I would go as soon as I noticed	68.8%	60.6%	73.5%	64.1%	55.1%	46.4%	43.3%	36.8%
Within 1 week	16.4%	19.7%	12.2%	14.9%	17.3%	21.0%	24.6%	23.2%
Between 1 and 2 weeks	10.6%	14.8%	9.2%	14.7%	17.6%	21.0%	20.2%	27.6%
Between 2 and 3 weeks	1.5%	2.0%	1.7%	1.7%	3.7%	5.0%	4.1%	4.7%
Between 3 and 4 weeks	0.7%	0.8%	0.9%	2.0%	2.0%	2.7%	2.3%	2.8%
More than a month	0.8%	1.0%	1.4%	1.8%	3.1%	2.6%	2.3%	2.3%
I would not contact my physician about this	0.6%	0.5%	0.4%	0.4%	0.7%	0.5%	1.9%	2.0%
I would contact another healthcare professional	0.0%	0.0%	0.1%		0.0%	0.1%	0.1%	0.1%
Does not respond	0.6%	0.6%	0.6%	0.4%	0.5%	0.7%	1.1%	0.5%
Chi^2^ test *p*-value		<0.001		<0.001		<0.001		<0.001

	Persistent unexplained pain		Change in the appearance of a mole		A sore that does not heal		Abdominal swelling	
	Wave		Wave		Wave		Wave	
	Pre	Post	Pre	Post	Pre	Post	Pre	Post
	*N* = 3269	*N* = 1500	*N* = 3269	*N* = 1500	*N* = 3269	*N* = 1500	*N* = 3269	*N* = 1500
I would go as soon as I noticed	41.3%	34.5%	42.7%	35.5%	33.8%	28.2%	29.4%	27.0%
Within 1 week	21.9%	22.9%	15.7%	15.2%	20.2%	19.4%	21.5%	19.0%
Between 1 and 2 weeks	22.2%	27.5%	19.2%	22.1%	27.1%	31.0%	25.5%	30.3%
Between 2 and 3 weeks	4.9%	6.8%	6.1%	7.8%	8.6%	10.2%	7.8%	8.0%
Between 3 and 4 weeks	3.1%	3.4%	5.1%	6.7%	4.2%	4.8%	4.2%	4.9%
More than a month	3.9%	3.0%	7.4%	7.6%	3.6%	3.8%	5.5%	5.6%
I would not contact my physician about this	1.6%	0.9%	2.9%	3.3%	1.8%	1.5%	5.0%	4.4%
I would contact another healthcare professional	0.1%	0.0%	0.0%	0.1%	0.1%	0.3%	0.1%	
Does not respond	1.0%	0.9%	1.0%	1.7%	0.6%	0.8%	1.1%	0.8%
Chi^2^ test *p*-value		<0.001		<0.001		.001		0.019

	Change in bowel or bladder habits		Unexplained weight loss		Persistent cough or hoarseness		Unexplained night sweats	
	Wave		Wave		Wave		Wave	
	Pre	Post	Pre	Post	Pre	Post	Pre	Post
	*N* = 3269	*N* = 1500	*N* = 3269	*N* = 1500	*N* = 3269	*N* = 1500	*N* = 3269	*N* = 1500
I would go as soon as I noticed	31.1%	26.0%	31.2%	25.5%	17.5%	17.3%	18.4%	15.2%
Within 1 week	18.3%	18.0%	11.7%	10.9%	17.0%	16.0%	15.4%	14.7%
Between 1 and 2 weeks	25.3%	29.9%	20.5%	24.5%	30.7%	28.5%	24.7%	27.5%
Between 2 and 3 weeks	8.6%	9.1%	10.1%	11.0%	9.9%	13.5%	8.8%	10.3%
Between 3 and 4 weeks	5.3%	7.8%	8.8%	12.7%	6.4%	9.9%	6.4%	9.5%
More than a month	6.4%	5.6%	11.3%	9.5%	9.6%	7.2%	9.8%	8.8%
I would not contact my physician about this	3.7%	2.7%	4.8%	4.4%	7.9%	6.5%	13.8%	11.1%
I would contact another healthcare professional	0.1%	0.3%	0.0%	0.1%	0.2%	0.2%	0.1%	0.4%
Does not respond	1.3%	0.6%	1.5%	1.3%	0.7%	1.1%	2.6%	2.4%
Chi^2^ test *p*-value		<0.001		<0.001		<0.001		0.001

	Unexplained tiredness	
	Wave	
	Pre	Post
	*N* = 3269	*N* = 1500
I would go as soon as I noticed	19.6%	16.8%
Within 1 week	14.1%	12.7%
Between 1 and 2 weeks	25.7%	29.5%
Between 2 and 3 weeks	10.5%	12.3%
Between 3 and 4 weeks	8.8%	11.0%
More than a month	11.7%	10.4%
I would not contact my physician about this	8.1%	6.3%
I would contact another healthcare professional	0.2%	0.0%
Does not respond	1.3%	1.0%
Chi^2^ test *p*-value		<0.001

The chi^2^ test tests for differences in the distribution of responses between the pre and post waves.

**Table 3:** Unadjusted (OR) and adjusted (adjOR) odds ratios for the effect of wave (post vs. pre) on help-seeking times as a function of the cut-off used (1, 2, or 3 weeks).

	Cut-off used to define a delayed response																							
	>1 week								>2 weeks								>3 weeks							
	OR	95% CI		*p*	adjOR	95% CI		*p*	OR	95% CI		*p*	adjOR	95% CI		*p*	OR	95% CI		*p*	adjOR		95% CI	*p*
Unexplained bleeding	1.44	1.22	1.69	<0.001	1.50	1.26	1.79	<0.001	1.42	0.91	1.69	0.170	1.24	0.89	1.74	0.206	1.06	0.71	1.58	0.784	1.08	0.70	1.67	0.736
Breast changes	1.63	1.31	2.03	<0.001	1.54	1.22	1.96	<0.001	1.31	0.91	1.90	0.152	1.15	0.77	1.73	0.504	1.56	1.00	2.44	0.053	1.27	0.77	2.10	0.351
Unexplained lump or swelling	1.26	1.10	1.44	0.001	1.25	1.08	1.45	0.003	1.16	0.95	1.42	0.143	1.22	0.99	1.52	0.066	1.01	0.78	1.31	0.939	1.03	0.77	1.36	0.860
Persistent difficulty in swallowing	1.44	1.27	1.64	<0.001	1.42	1.24	1.64	<0.001	1.11	0.92	1.35	0.283	1.09	0.88	1.34	0.438	1.07	0.84	1.36	0.604	1.04	0.80	1.35	0.780
Persistent unexplained pain	1.29	1.13	1.46	<0.001	1.28	1.11	1.47	<0.001	1.06	0.89	1.27	0.494	1.09	0.91	1.32	0.360	0.85	0.68	1.07	0.167	0.82	0.64	1.06	0.126
Change in the appearance of a mole	1.34	1.18	1.52	<0.001	1.32	1.15	1.52	<0.001	1.25	1.09	1.45	0.002	1.22	1.04	1.43	0.014	1.18	1.00	1.39	0.050	1.13	0.94	1.35	0.183
A sore that does not heal	1.28	1.13	1.45	<0.001	1.23	1.08	1.42	0.003	1.15	0.99	1.10	0.074	1.10	0.93	1.30	0.270	1.07	0.87	1.31	0.546	1.01	0.81	1.26	0.951
Abdominal swelling	1.22	1.08	1.39	0.001	1.18	1.03	1.35	0.018	1.01	0.88	1.18	0.809	1.03	0.88	1.21	0.712	1.01	0.85	1.21	0.874	1.00	0.83	1.21	0.980
Change in bowel or bladder habits	1.25	1.11	1.42	<0.001	1.27	1.10	1.46	0.001	1.06	0.92	1.22	0.421	1.04	0.89	1.22	0.600	1.05	0.89	1.24	0.599	0.97	0.81	1.17	0.754
Unexplained weight loss	1.32	1.16	1.50	<0.001	1.34	1.16	1.54	<0.001	1.12	0.98	1.27	0.087	1.09	0.95	1.26	0.232	1.09	0.95	1.26	0.208	1.05	0.90	1.22	0.564
Persistent cough or hoarseness	1.06	0.93	1.20	0.420	1.09	0.95	1.26	0.214	1.16	1.01	1.32	0.026	0.99	0.85	1.14	0.860	0.98	0.85	1.13	0.818	0.94	0.80	1.10	0.421
Unexplained night sweats	1.19	1.04	1.36	0.010	1.19	1.03	1.38	0.022	1.04	0.92	1.18	0.545	1.04	0.90	1.19	0.606	0.97	0.85	1.11	0.961	0.99	0.85	1.14	0.845
Unexplained tiredness	1.23	1.08	1.40	0.002	1.20	1.03	1.39	0.019	1.03	0.91	1.17	0.597	0.98	0.85	1.13	0.773	0.94	0.82	1.08	0.410	0.91	0.78	1.06	0.207

Adjusted ORs are from models adjusted for age, sex, socioeconomic position, civil status, personal cancer history and having a close family member diagnosed with cancer. ORs refer to the odds of reporting waiting for more than the time specified as the cut-off in the post- compared to the pre-wave.

Delay scores were higher for respondents who were male, younger, from a higher socioeconomic position, single (vs. married and separated/divorced), and had a close family member diagnosed with cancer (Supplementary Tables S1 and S2). There was an effect of wave, which was different depending on sex and age (Table 4).

**Table 4:** Relative score increases (RSI) for the effect of wave in different demographic groups derived from multiple Poisson regression analyses with interaction terms on total delay scores and total barrier scores.

	Delay scores				Barrier scores			
		95% CI				95% CI		
	RSI	Lower	Upper	*p*	RSI	Lower	Upper	*p*
Sex								
Males	1.07	1.03	1.11	0.001	1.18	1.08	1.30	<0.001
Females	1.14	1.10	1.18	<0.001	1.34	1.24	1.45	<0.001
Age								
18–24	1.02	0.93	1.11	0.673	1.17	0.96	1.41	0.114
25–34	1.08	1.01	1.15	0.025	1.13	0.98	1.31	0.084
35–44	1.12	1.06	1.19	<0.001	1.21	1.06	1.39	0.006
45–54	1.05	0.99	1.12	0.123	1.19	1.04	1.37	0.013
55–64	1.21	1.13	1.30	<0.001	1.30	1.10	1.53	0.002
65+	1.17	1.10	1.25	<0.001	1.73	1.49	1.99	<0.001

Delay scores increased from pre to post more strongly for women than for men (see also Supplementary Fig. S1). Looking at the different symptoms, the pre–post difference in the percentage of respondents who would seek help within a week of symptom onset was larger for women than for men for all symptoms with the exception of persistent difficulty in swallowing (Supplementary Fig. S2). The most pronounced difference was found for changes in the appearance of a mole: in the pre-wave 65% of women said they would seek help within a week and this number fell to 55% in the post wave, whereas for men the difference was much smaller (a drop of 50% to 48% pre–post, respectively).

Regarding age, delay scores increased significantly from pre to post for all groups, except for 18–24 and 45–54 years. The increase was largest among the two oldest groups (55–64 and 65+) (see also Supplementary Fig. S3).

The results with the alternative delays scores were generally similar (Supplementary Tables S3 and S4). Because only women were asked regarding breast changes, we also conducted analyses not taking this question into account in the delay scores: it did not change the general pattern of results or the interaction between wave and sex (Supplementary Tables S5 and S6).

### Perceived barriers to help-seeking

**Fig. 2:**
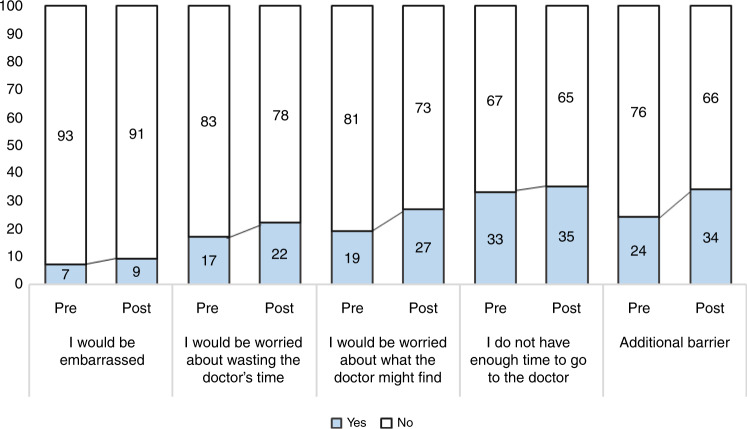
Perceived barriers as a function of wave. Percentage of respondents reporting different barriers to help-seeking for symptoms as a function of wave (pre vs. post).

Respondents were significantly more likely to report all but one barrier in the post compared to the pre-wave (Fig. 2). The largest increases were observed in reporting an additional barrier (adjOR = 1.66, 95% CI 1.44–1.92, *p* < 0.001), followed by worry about what the doctor may find (adjOR = 1.58, 95% CI 1.35–1.84, *p* < 0.001), being worried about wasting the doctor’s time (adjOR = 1.48, 95% CI 1.25–1.74, *p* < 0.001), and being embarrassed (adjOR = 1.42, 95% CI 1.11–1.81, *p* = 0.005). The additional barriers reported included not perceiving the symptoms as important enough (10% vs. 13% in pre- vs post-wave, respectively), family or work obligations (6% vs. 7%), other diverse issues (4% vs. 8%), barriers related to the functioning of the health system (4% in both waves), and the coronavirus (0% vs. 2%).

More barriers were reported by respondents who were female, younger, separated or divorced (vs. married), and had a close family member diagnosed with cancer (Supplementary Table S2). There was an effect of wave that was different depending on sex and age (Table 4). Generally, the number of reported barriers increased from pre to post and this increase was more pronounced for women (Supplementary Fig. S1) and older respondents (Supplementary Fig. S3).

Looking at the different barriers, the percentage of respondents who endorsed each barrier increased from pre to post among both men and women, but this increase was notably larger in women (Supplementary Table S7). There was one exception: the barrier ‘not having enough time to go to the doctor’ increased among women (32% to 37%) but decreased slightly among men (34% to 32%).

**Fig. 3:**
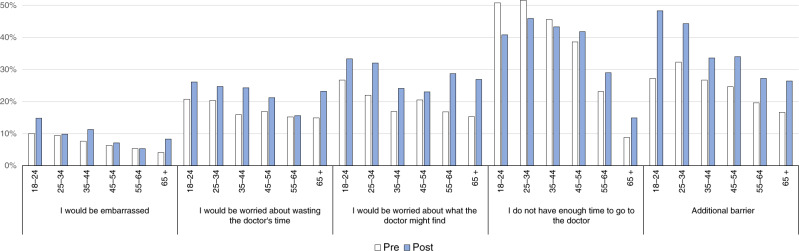
Perceived barriers according to age group. Percentage of respondents reporting different barriers to help-seeking for symptoms as a function of wave (pre vs. post) and age group.

Older respondents (e.g. 65+) reported consistent increases from pre to post on all barriers, especially worry about wasting the doctor’s time and worry about what the doctor may find (Fig. 3). In contrast, whereas most barriers increased among younger respondents (18–44) from pre to post, the percentage of respondents who reported not having enough time to go to the doctor decreased in this group.

## Discussion

We observed a consistent increase in anticipated help-seeking times for 12 out of 13 cancer symptoms comparing answers registered before the coronavirus pandemic was announced to those gathered 5 months after. In particular, the odds of seeking care later than a week after symptom onset increased by about 20–50%, with the largest increases for breast changes and unexplained bleeding. This should be a matter of concern because breast changes and unexplained bleeding are warning signs of the most incident cancers in the Spanish population (breast cancer in females, and colorectal cancer in both sexes, respectively).^20^

The changes observed are not large in absolute terms (e.g. 4–9% average increases in the percentage of people who would not seek help within a week, Fig. 1). However, they would only add further delays to those produced on the other intervals of the cancer care pathway (e.g. waiting times until the appointment with the physician, referral, diagnostic investigations, and treatment scheduling delays).^9,21^

The only symptom for which no increase was observed was persistent cough or hoarseness. The reason may be related to the pandemic, since the population is aware that cough is also a symptom of COVID-19, and this would speed up help-seeking. Actually, using 4-weeks as a cut-off for this symptom following previous studies,^18^ we observed a decrease in the number of people who would wait beyond this period (17.5% pre to 13.7% post).

Given the lack of official guidelines regarding the maximum time recommended to wait before seeking help for symptoms compatible with cancer, previous research has used a variety of cut-offs to define delayed responses (ranging from 1 to 4 weeks), based on the distribution of responses or the urgency of symptoms. For this reason, we conducted sensitivity analyses using different cut-offs (reported in the Supplement). Although the global scores based on the 2-week cut-off reliably detected the pre–post-pandemic changes, as can be seen in Fig. 1, pre–post differences were most pronounced considering the cut-off of 1 week. This shows that the documented increases in the time it would take to seek help are not large in terms of the number of days or weeks most individuals would wait.

However, we believe they are of the highest concern because anticipated help-seeking times reported in general population surveys tend to be very optimistic in comparison to real help-seeking times reported by patients who experienced symptoms. Whereas the majority of survey participants report they would seek help within a week of symptom onset for most cancer symptoms, it is not uncommon for cancer patients to report having waited more than a month to consult with a healthcare professional.^22^ For instance, in the study by Lyratzopoulos, the proportion of patients who waited more than a month was at least 25% for 22 out of 28 cancers.^23^

Respondents were generally more likely to report barriers 5 months after the pandemic started, suggesting that there may be a general perception of lower availability of healthcare services. In the post-wave, more respondents were worried about wasting the doctor’s time, suggesting that in the current circumstances many people may perceive that they should seek care only if absolutely necessary. However, the percentage of people who reported worry about what the doctor may find also increased in the post wave, suggesting that there could be an overall increase in health-related concern in the general population. These perceived barriers should be investigated further and addressed in the population because they are important drivers of help-seeking.^16,18^ Interestingly, only 2% of respondents mentioned the coronavirus as a barrier to help-seeking and it was not singled out as the main reason for delays in help-seeking.

In the pre-wave, women reported faster help-seeking times than men, consistent with the documented higher reluctance of men to consult for diverse health problems.^24^ However, this advantage disappeared in the post wave, where both women’s delay and barrier scores increased to a larger extent than men’s. If this pattern persists, in the long-term women’s cancer outcomes might be disproportionately negatively affected by the pandemic situation. The analysis of specific symptoms showed that pre–post differences for women were especially pronounced for the skin cancer symptom (changes in the appearance of a mole), suggesting that women could be at high risk of delayed diagnosis for this cancer. Additional delays and barriers to help-seeking may be a result of the larger burden of the pandemic on the female population^25^ in terms of coping with work and family obligations, which emerge as competing goals.^26^ This supposition is supported by the finding that the percentage of women who reported not having time to go to the doctor increased in the post wave, whereas it decreased in men.

An encouraging finding is that consistent with previous research,^16,18,27^ the population with the highest cancer incidence (older adults +65 years old), reported the fastest help-seeking times and the fewest barriers. However, both help-seeking times and perceived barriers increased to a larger extent in this group (e.g. a median of 0 barriers in the pre vs. median of 1 in the post wave).

The increase in help-seeking times for younger and middle-aged adults is also worrying because recent modelling studies show that it is precisely younger patients who would be most affected by the COVID-19-related delays in the cancer care pathway.^28^ In particular, survival decrements for even small diagnostic delays are expected to be substantial for most tumours in individuals under 70.^28^

To the best of our knowledge, this is the first population-based survey to investigate anticipated help-seeking for cancer symptoms in Spain. Despite the increases documented after the start of the pandemic, anticipated help-seeking times in Spain remain generally prompt and similar (e.g. Australia, Canada, Sweden) or notably quicker (e.g. UK and Denmark) than those documented in high-income countries of the ICBP.^16,18^ The exception is breast changes, with post-pandemic help-seeking times (20% seeking help >1 week) notably longer than those documented in all but one country of the ICBP.^18^ This is especially worrying due to the suspension of the organised breast screening programs in many Spanish regions. In contrast, in comparison to residents of the other ICBP countries (with the exception of the UK),^29^ Spaniards report more barriers to help-seeking.

Limitations of this research include potential selection biases related to survey non-response, a smaller sample in the post compared to the pre-wave, and some differences in the socio-demographic characteristics of the respondents in the two waves. The pre-wave data collection took place immediately before the state of emergency was announced when the coronavirus was already a serious problem in Spain. Thus, it is possible that the reported help-seeking times and barriers in the pre-wave may already have been affected by the coronavirus crisis.

It is not clear to what extent anticipated help-seeking times are indicative of actual help-seeking times for experienced symptoms, although results tend to be similar across studies regarding drivers of help-seeking.^30^ We used the international ABC questionnaire; however, it was not previously validated for use in Spain or any other Southern-European or Spanish-speaking country, so measurement problems may exist. In addition, because it was not created specifically for the Spanish culture and health system, we had to adapt some answer options and included an open-ended question about barriers to capture issues beyond the predefined questionnaire. For instance, competing family and work obligations emerged as an additional frequent barrier to help-seeking that was not considered in the original questionnaire.

The results of this population survey in Spain provide the first evidence in support of the anticipated increases in help-seeking times for cancer symptoms during the coronavirus pandemic.^2,8,9^ The results suggest that there is an urgent need for interventions encouraging people to consult their physicians with symptoms suggestive of cancer and addressing the barriers that have become most pronounced during the pandemic situation, especially among women and older individuals.^9,13^

## Supplementary information

Supplement

## Data Availability

The dataset used for the current study can be requested from the Spanish Association against Cancer (Asociación Española Contra el Cáncer: www.aecc.es).
