# Supplementary material for: Anticipated help-seeking for cancer symptoms before and after the coronavirus pandemic: results from the Onco-barometer population survey in Spain
Source: Br J Cancer. 2021 Apr 14;124(12):2017–25. doi: 10.1038/s41416-021-01382-1 (PMC8044659; doi:10.1038/s41416-021-01382-1)
Supplement: Supplementary file 1 — Supplement [file 41416_2021_1382_MOESM1_ESM.docx]

Table S1. Total delay and barrier scores as a function of socio-demographic characteristics and wave (Pre vs. Post).

|  |  |  | Pre-Coronavirus | | | | Post-Coronavirus | | | |
| --- | --- | --- | --- | --- | --- | --- | --- | --- | --- | --- |
|  |  | | Total delay score (1-week cut-off) | Total delay score (2-week cut-off) | Total delay score (3-week cut-off) | Total barrier score | Total delay score (1-week cut-off) | Total delay score (2-week cut-off) | Total delay score (3-week cut-off) | Total barrier score |
| Sex | Male | Mean | 5.82 | 2.92 | 1.97 | 0.96 | 6.29 | 2.93 | 1.87 | 1.16 |
|  |  | SD | 3.75 | 3.04 | 2.55 | 1.02 | 3.82 | 3.00 | 2.50 | 1.06 |
|  |  | Median | 6.00 | 2.00 | 1.00 | 1.00 | 7.00 | 2.00 | 1.00 | 1.00 |
|  | Female | Mean | 5.24 | 2.59 | 1.81 | 1.00 | 6.03 | 2.93 | 1.95 | 1.36 |
|  |  | SD | 3.75 | 2.90 | 2.44 | 1.07 | 3.83 | 3.06 | 2.51 | 1.14 |
|  |  | Median | 5.00 | 2.00 | 1.00 | 1.00 | 6.00 | 2.00 | 1.00 | 1.00 |
| Age | 18-24 | Mean | 6.92 | 3.71 | 2.45 | 1.36 | 7.09 | 3.00 | 1.94 | 1.63 |
|  |  | SD | 3.19 | 2.89 | 2.49 | 1.07 | 3.46 | 2.82 | 2.20 | 1.16 |
|  |  | Median | 7.00 | 4.00 | 2.00 | 1.00 | 7.00 | 2.00 | 1.00 | 2.00 |
|  | 25-34 | Mean | 6.81 | 3.65 | 2.47 | 1.36 | 7.42 | 3.85 | 2.54 | 1.56 |
|  |  | SD | 3.44 | 3.06 | 2.67 | 1.11 | 3.41 | 3.06 | 2.61 | 1.05 |
|  |  | Median | 7.00 | 3.00 | 2.00 | 1.00 | 8.00 | 4.00 | 2.00 | 1.49 |
|  | 35-44 | Mean | 6.16 | 3.12 | 2.10 | 1.12 | 6.76 | 3.43 | 2.26 | 1.36 |
|  |  | SD | 3.71 | 3.09 | 2.57 | 1.07 | 3.95 | 3.24 | 2.83 | 1.07 |
|  |  | Median | 6.00 | 2.78 | 1.00 | 1.00 | 7.00 | 3.00 | 1.00 | 1.00 |
|  | 45-54 | Mean | 5.67 | 2.81 | 2.03 | 1.07 | 6.30 | 3.11 | 2.01 | 1.27 |
|  |  | SD | 3.79 | 3.11 | 2.69 | 1.10 | 3.64 | 3.16 | 2.68 | 1.15 |
|  |  | Median | 6.00 | 2.00 | 1.00 | 1.00 | 7.00 | 2.00 | 1.00 | 1.00 |
|  | 55-64 | Mean | 4.83 | 2.25 | 1.51 | 0.80 | 5.93 | 2.73 | 1.66 | 1.06 |
|  |  | SD | 3.61 | 2.72 | 2.27 | 0.95 | 4.03 | 3.04 | 2.33 | 1.03 |
|  |  | Median | 4.00 | 1.00 | 1.00 | 1.00 | 6.00 | 2.00 | 1.00 | 1.00 |
|  | 65 + | Mean | 4.02 | 1.85 | 1.30 | 0.60 | 4.49 | 1.88 | 1.31 | 0.99 |
|  |  | SD | 3.61 | 2.52 | 2.10 | 0.85 | 3.55 | 2.43 | 2.03 | 1.07 |
|  |  | Median | 3.00 | 1.00 | 0.00 | 0.00 | 4.00 | 1.00 | 0.00 | 1.00 |
| SEP | Group 1 | Mean | 6.91 | 3.54 | 2.32 | 1.04 | 7.58 | 3.90 | 2.37 | 1.28 |
|  |  | SD | 3.70 | 3.21 | 2.65 | 1.03 | 3.71 | 3.04 | 2.34 | 1.04 |
|  |  | Median | 7.00 | 3.00 | 1.00 | 1.00 | 8.00 | 4.00 | 2.00 | 1.00 |
|  | Group 2 | Mean | 6.24 | 3.23 | 2.19 | 1.04 | 6.59 | 3.24 | 2.02 | 1.22 |
|  |  | SD | 3.71 | 3.17 | 2.78 | 1.03 | 3.76 | 3.10 | 2.69 | 1.04 |
|  |  | Median | 6.00 | 2.00 | 1.00 | 1.00 | 7.00 | 2.00 | 1.00 | 1.00 |
|  | Group 3 | Mean | 6.13 | 2.95 | 2.01 | 1.00 | 6.91 | 3.26 | 2.12 | 1.27 |
|  |  | SD | 3.73 | 2.93 | 2.52 | 1.05 | 3.69 | 3.13 | 2.58 | 1.08 |
|  |  | Median | 6.00 | 2.00 | 1.00 | 1.00 | 7.00 | 2.00 | 1.00 | 1.00 |
|  | Group 4+5 | Mean | 4.94 | 2.42 | 1.55 | 0.84 | 6.09 | 2.65 | 1.69 | 1.20 |
|  |  | SD | 3.70 | 2.78 | 2.12 | 0.93 | 3.74 | 2.88 | 2.14 | 1.22 |
|  |  | Median | 5.00 | 1.00 | 1.00 | 1.00 | 6.08 | 2.00 | 1.00 | 1.00 |
|  | Group 6 | Mean | 4.85 | 2.36 | 1.70 | 1.05 | 5.16 | 2.44 | 1.63 | 1.28 |
|  |  | SD | 3.55 | 2.72 | 2.32 | 1.10 | 3.82 | 3.08 | 2.51 | 1.17 |
|  |  | Median | 5.00 | 1.00 | 1.00 | 1.00 | 5.00 | 1.00 | 0.00 | 1.00 |
|  | Group 7 | Mean | 4.15 | 2.08 | 1.44 | 0.86 | 5.40 | 2.55 | 1.73 | 1.35 |
|  |  | SD | 3.49 | 2.73 | 2.26 | 1.08 | 3.80 | 2.76 | 2.43 | 1.10 |
|  |  | Median | 4.00 | 1.00 | 0.00 | 1.00 | 5.00 | 2.00 | 0.63 | 1.00 |
| Civil status | Married or cohabiting | Mean | 5.30 | 2.59 | 1.77 | 0.90 | 5.89 | 2.71 | 1.76 | 1.16 |
|  |  | SD | 3.71 | 2.92 | 2.41 | 1.00 | 3.83 | 2.97 | 2.47 | 1.09 |
|  |  | Median | 5.00 | 2.00 | 1.00 | 1.00 | 6.00 | 2.00 | 1.00 | 1.00 |
|  | Single | Mean | 6.35 | 3.30 | 2.27 | 1.18 | 6.94 | 3.39 | 2.17 | 1.48 |
|  |  | SD | 3.66 | 3.09 | 2.68 | 1.10 | 3.69 | 3.05 | 2.54 | 1.14 |
|  |  | Median | 7.00 | 3.00 | 1.00 | 1.00 | 7.00 | 3.00 | 1.00 | 1.00 |
|  | Separated or divorced | Mean | 5.11 | 2.53 | 1.72 | 1.06 | 5.55 | 2.92 | 2.02 | 1.28 |
|  |  | SD | 3.74 | 2.82 | 2.40 | 1.07 | 3.79 | 3.24 | 2.73 | 1.03 |
|  |  | Median | 5.00 | 2.00 | 1.00 | 1.00 | 5.00 | 2.00 | 1.00 | 1.00 |
|  | Widowed | Mean | 3.50 | 1.59 | 1.17 | 0.64 | 4.63 | 2.09 | 1.49 | 0.94 |
|  |  | SD | 3.51 | 2.34 | 2.01 | 0.95 | 3.71 | 2.70 | 2.21 | 0.92 |
|  |  | Median | 3.00 | 1.00 | 0.00 | 0.00 | 5.00 | 1.00 | 1.00 | 1.00 |
| Personal cancer history | No | Mean | 5.58 | 2.80 | 1.92 | 1.01 | 6.26 | 2.98 | 1.94 | 1.29 |
|  |  | SD | 3.76 | 3.00 | 2.52 | 1.06 | 3.80 | 3.04 | 2.52 | 1.11 |
|  |  | Median | 6.00 | 2.00 | 1.00 | 1.00 | 6.00 | 2.00 | 1.00 | 1.00 |
|  | Yes | Mean | 4.48 | 2.04 | 1.42 | 0.68 | 5.07 | 2.40 | 1.63 | 0.99 |
|  |  | SD | 3.55 | 2.39 | 2.05 | 0.89 | 3.95 | 2.90 | 2.34 | 1.02 |
|  |  | Median | 4.00 | 1.00 | 1.00 | 0.00 | 4.00 | 1.00 | 1.00 | 1.00 |
| Family member with cancer | No | Mean | 5.13 | 2.47 | 1.64 | 0.92 | 5.88 | 2.64 | 1.62 | 1.19 |
|  |  | SD | 3.74 | 2.80 | 2.28 | 1.05 | 3.76 | 2.92 | 2.25 | 1.11 |
|  |  | Median | 5.00 | 1.00 | 1.00 | 1.00 | 6.00 | 2.00 | 1.00 | 1.00 |
|  | Yes | Mean | 5.61 | 2.83 | 1.97 | 1.01 | 6.24 | 3.02 | 2.01 | 1.29 |
|  |  | SD | 3.75 | 3.01 | 2.55 | 1.05 | 3.85 | 3.06 | 2.58 | 1.11 |
|  |  | Median | 6.00 | 2.00 | 1.00 | 1.00 | 7.00 | 2.00 | 1.00 | 1.00 |

Note: SEP= Socioeconomic position: GROUP 1. Directors and managers of establishments with 10 or more employees and professionals traditionally associated with university degrees. GROUP 2. Directors and managers of establishments with fewer than 10 employees and professionals traditionally associated with university degrees. GROUP 3. Intermediate occupations: employees of the administrative type and professionals supporting administrative management. GROUP 4+5. Free-lancers/self-employed. Supervisors and workers in qualified technical occupations. GROUP 6. Qualified workers of the primary sector and other semi-qualified workers. Group 7. Unskilled workers.

Table S2. Model main effects and Relative Score Increases (RSI) from multiple Poisson regression analyses on total delay (cut-off 1 week) and barrier scores.

|  |  | **Delay scores** | | | | **Barrier scores** | | | |
| --- | --- | --- | --- | --- | --- | --- | --- | --- | --- |
|  | Parameter | RSI | 95% CI | |  | RSI | 95% CI | |  |
|  |  |  | Lower | Upper | p |  | Lower | Upper | p |
|  | (Intercept)* | 2.06 | 1.99 | 2.13 | 0.000 | 0.10 | -0.06 | 0.25 | 0.232 |
| Wave | Post vs. Pre | 1.11 | 1.08 | 1.14 | 0.000 | 1.27 | 1.20 | 1.35 | <0.001 |
| Sex | Female vs. Male | 0.97 | 0.94 | 0.99 | 0.012 | 1.13 | 1.06 | 1.20 | <0.001 |
| Age | 65+ vs. 18-24 | 0.65 | 0.61 | 0.69 | 0.000 | 0.55 | 0.48 | 0.63 | <0.001 |
|  | 55-64 vs. 18-24 | 0.76 | 0.72 | 0.81 | 0.000 | 0.63 | 0.55 | 0.72 | <0.001 |
|  | 45-54 vs. 18-24 | 0.85 | 0.80 | 0.90 | 0.000 | 0.81 | 0.71 | 0.92 | 0.001 |
|  | 35-44 vs. 18-24 | 0.90 | 0.85 | 0.95 | 0.000 | 0.87 | 0.77 | 0.98 | 0.020 |
|  | 25-34 vs. 18-24 | 0.96 | 0.91 | 1.01 | 0.132 | 1.01 | 0.90 | 1.13 | 0.866 |
| SEP | Group 7 vs. 1 | 0.69 | 0.65 | 0.72 | 0.000 | 0.99 | 0.88 | 1.11 | 0.846 |
|  | Group 6 vs. 1 | 0.71 | 0.68 | 0.74 | 0.000 | 1.03 | 0.94 | 1.14 | 0.501 |
|  | Group 4+5 vs. 1 | 0.79 | 0.75 | 0.84 | 0.000 | 0.95 | 0.84 | 1.08 | 0.440 |
|  | Group 3 vs. 1 | 0.91 | 0.87 | 0.94 | 0.000 | 0.98 | 0.89 | 1.09 | 0.723 |
|  | Group 2 vs. 1 | 0.90 | 0.86 | 0.94 | 0.000 | 0.99 | 0.89 | 1.11 | 0.914 |
| Civil status | Widowed vs. Married | 0.86 | 0.81 | 0.91 | 0.000 | 0.95 | 0.82 | 1.10 | 0.470 |
|  | Sep./divorced vs. Married | 1.00 | 0.95 | 1.05 | 0.925 | 1.15 | 1.03 | 1.28 | 0.014 |
|  | Single vs. Married | 1.04 | 1.01 | 1.08 | 0.009 | 1.07 | 0.99 | 1.15 | 0.074 |
| Personal cancer history | Yes vs. No | 0.97 | 0.93 | 1.02 | 0.203 | 0.92 | 0.82 | 1.03 | 0.146 |
| Close family member with cancer | Yes vs. No | 1.07 | 1.04 | 1.10 | 0.000 | 1.09 | 1.02 | 1.17 | 0.013 |

Note: * Mean number of symptoms with delayed response or reported barriers adjusted for the remaining factors in the reference group. CI= confidence intervals; Socioeconomic position: GROUP 1. Directors and managers of establishments with 10 or more employees and professionals traditionally associated with university degrees. GROUP 2. Directors and managers of establishments with fewer than 10 employees and professionals traditionally associated with university degrees. GROUP 3. Intermediate occupations: employees of the administrative type and professionals supporting administrative management. GROUP 4+5 (merged due to small sample size). Free-lancers/self-employed. Supervisors and workers in qualified technical occupations. GROUP 6. Qualified workers of the primary sector and other semi-qualified workers. Group 7. Unskilled workers.

Table S3. Model main effects and Relative Score Increases (RSI) from multiple Poisson regression analyses on total delay scores with alternative cut-offs (2 and 3 weeks).

|  |  | **Delay scores (2-week cut-off)** | | | | **Delay scores (3-week cut-off)** | | | |
| --- | --- | --- | --- | --- | --- | --- | --- | --- | --- |
|  |  | RSI | 95% CI | |  | RSI | 95% CI | |  |
|  |  |  | Lower | Upper | p |  | Lower | Upper | p |
|  | (Intercept)* | 1.35 | 1.25 | 1.44 | <.001 | 0.79 | 0.67 | 0.90 | .000 |
| Wave | Post vs. Pre | 1.06 | 1.02 | 1.10 | 0.005 | 0.99 | 0.94 | 1.04 | 0.710 |
| Sex | Female vs. Male | 0.97 | 0.93 | 1.00 | 0.074 | 0.98 | 0.94 | 1.03 | 0.440 |
| Age | 65+ vs. 18-24 | 0.60 | 0.55 | 0.65 | <.001 | 0.66 | 0.59 | 0.73 | 0.000 |
|  | 55-64 vs. 18-24 | 0.71 | 0.66 | 0.78 | <.001 | 0.74 | 0.67 | 0.82 | 0.000 |
|  | 45-54 vs. 18-24 | 0.86 | 0.80 | 0.93 | <.001 | 0.95 | 0.86 | 1.05 | 0.333 |
|  | 35-44 vs. 18-24 | 0.92 | 0.85 | 0.99 | 0.033 | 0.98 | 0.90 | 1.08 | 0.745 |
|  | 25-34 vs. 18-24 | 1.04 | 0.96 | 1.12 | 0.339 | 1.10 | 1.00 | 1.20 | 0.039 |
| SEP | Group 7 vs. 1 | 0.67 | 0.62 | 0.72 | <.001 | 0.70 | 0.64 | 0.77 | 0.000 |
|  | Group 6 vs. 1 | 0.67 | 0.63 | 0.71 | <.001 | 0.74 | 0.69 | 0.80 | 0.000 |
|  | Group 4+5 vs. 1 | 0.75 | 0.70 | 0.81 | <.001 | 0.75 | 0.69 | 0.82 | 0.000 |
|  | Group 3 vs. 1 | 0.85 | 0.80 | 0.90 | <.001 | 0.88 | 0.82 | 0.95 | 0.001 |
|  | Group 2 vs. 1 | 0.90 | 0.85 | 0.96 | 0.001 | 0.93 | 0.86 | 1.00 | 0.043 |
| Civil status | Widowed vs. Married | 0.88 | 0.80 | 0.97 | 0.001 | 0.91 | 0.81 | 1.01 | 0.081 |
|  | Sep./divorced vs. Married | 1.07 | 0.99 | 1.14 | 0.073 | 1.10 | 1.02 | 1.20 | 0.020 |
|  | Single vs. Married | 1.08 | 1.03 | 1.13 | 0.001 | 1.10 | 1.04 | 1.16 | 0.001 |
| Personal cancer history | Yes vs. No | 0.96 | 0.90 | 1.03 | 0.239 | 0.97 | 0.89 | 1.06 | 0.513 |
| Close family member with cancer | Yes vs. No | 1.12 | 1.08 | 1.17 | <.001 | 1.20 | 1.14 | 1.26 | 0.000 |

Note: *Mean number of symptoms with delayed response adjusted for the remaining factors. CI= confidence intervals; Socioeconomic position: GROUP 1. Directors and managers of establishments with 10 or more employees and professionals traditionally associated with university degrees. GROUP 2. Directors and managers of establishments with fewer than 10 employees and professionals traditionally associated with university degrees. GROUP 3. Intermediate occupations: employees of the administrative type and professionals supporting administrative management. GROUP 4+5 (merged due to small sample size). Free-lancers/self-employed. Supervisors and workers in qualified technical occupations. GROUP 6. Qualified workers of the primary sector and other semi-qualified workers. Group 7. Unskilled workers.

Table S4. Relative Score Increases (RSI) for the effect of wave in different demographic groups derived from multiple Poisson regression analyses with interaction terms on total delay scores with alternative cut-offs (2 and 3 weeks).

|  |  | **Cut-off 2 weeks** | | | | **Cut-off 3 weeks** | | | |
| --- | --- | --- | --- | --- | --- | --- | --- | --- | --- |
|  |  | RSI | 95% CI | |  | RSI | 95% CI | |  |
|  |  |  | Lower | Upper | p |  | Lower | Upper | p |
| Sex | Males | 0.99 | 0.94 | 1.05 | 0.856 | 0.90 | 0.84 | 0.97 | 0.006 |
|  | Females | 1.12 | 1.06 | 1.18 | <.001 | 1.06 | 1.00 | 1.13 | 0.080 |
| Age | 18-24 | 0.80 | 0.70 | 0.91 | 0.001 | 0.78 | 0.66 | 0.92 | 0.004 |
|  | 25-34 | 1.05 | 0.96 | 1.15 | 0.290 | 1.06 | 0.95 | 1.19 | 0.304 |
|  | 35-44 | 1.13 | 1.03 | 1.22 | 0.006 | 1.09 | 0.98 | 1.21 | 0.100 |
|  | 45-54 | 1.07 | 0.98 | 1.16 | 0.154 | 0.92 | 0.83 | 1.02 | 0.129 |
|  | 55-64 | 1.19 | 1.07 | 1.32 | 0.001 | 1.07 | 0.94 | 1.22 | 0.307 |
|  | 65+ | 1.04 | 0.94 | 1.15 | 0.422 | 1.02 | 0.90 | 1.14 | 0.794 |

Table S5. Model main effects and relative score increases (RSI) from multiple Poisson regression analyses on total delay scores without considering the variable breast changes.

|  |  | **Delay scores (1-week cut-off)** | | | | **Delay scores (2-week cut-off)** | | | | **Delay scores (3-week cut-off)** | | | |
| --- | --- | --- | --- | --- | --- | --- | --- | --- | --- | --- | --- | --- | --- |
|  |  | RSI | 95% CI | |  | RSI | 95% CI | |  | RSI | 95% CI | |  |
|  |  |  | Lower | Upper | p |  | Lower | Upper | p |  | Lower | Upper | p |
|  | (Intercept)* | 2.05 | 1.99 | 2.12 | <0.001 | 1.34 | 1.25 | 1.44 | <0.001 | 0.79 | 0.67 | 0.91 | .000 |
| Wave | Post vs. Pre | 1.10 | 1.07 | 1.13 | <0.001 | 1.05 | 1.01 | 1.09 | 0.010 | 0.99 | 0.94 | 1.04 | 0.644 |
| Sex | Female vs. Male | 0.94 | 0.92 | 0.96 | <0.001 | 0.95 | 0.91 | 0.98 | 0.004 | 0.97 | 0.92 | 1.01 | 0.135 |
| Age | 65+ vs. 18-24 | 0.65 | 0.61 | 0.69 | <0.001 | 0.60 | 0.55 | 0.66 | <0.001 | 0.66 | 0.59 | 0.73 | <0.001 |
|  | 55-64 vs. 18-24 | 0.76 | 0.72 | 0.81 | <0.001 | 0.72 | 0.66 | 0.78 | <0.001 | 0.74 | 0.66 | 0.82 | <0.001 |
|  | 45-54 vs. 18-24 | 0.85 | 0.81 | 0.90 | <0.001 | 0.87 | 0.80 | 0.94 | 0.001 | 0.96 | 0.87 | 1.05 | 0.373 |
|  | 35-44 vs. 18-24 | 0.90 | 0.85 | 0.95 | <0.001 | 0.94 | 0.87 | 1.01 | 0.092 | 0.99 | 0.90 | 1.08 | 0.790 |
|  | 25-34 vs. 18-24 | 0.97 | 0.92 | 1.02 | 0.190 | 1.05 | 0.98 | 1.13 | 0.196 | 1.10 | 1.01 | 1.21 | 0.037 |
| SEP | Group 7 vs. 1 | 0.69 | 0.66 | 0.73 | <0.001 | 0.66 | 0.62 | 0.71 | <0.001 | 0.71 | 0.65 | 0.77 | <0.001 |
|  | Group 6 vs. 1 | 0.71 | 0.68 | 0.74 | <0.001 | 0.67 | 0.63 | 0.71 | <0.001 | 0.74 | 0.69 | 0.80 | <0.001 |
|  | Group 4+5 vs. 1 | 0.80 | 0.76 | 0.84 | <0.001 | 0.75 | 0.69 | 0.80 | <0.001 | 0.76 | 0.69 | 0.83 | <0.001 |
|  | Group 3 vs. 1 | 0.91 | 0.87 | 0.95 | <0.001 | 0.84 | 0.79 | 0.89 | <0.001 | 0.88 | 0.82 | 0.95 | 0.001 |
|  | Group 2 vs. 1 | 0.90 | 0.86 | 0.94 | <0.001 | 0.89 | 0.84 | 0.95 | <0.001 | 0.92 | 0.86 | 1.00 | 0.038 |
| Civil status | Widowed vs. Married | 0.86 | 0.81 | 0.92 | <0.001 | 0.88 | 0.80 | 0.96 | 0.006 | 0.91 | 0.81 | 1.01 | 0.084 |
|  | Sep./divorced vs. Married | 1.00 | 0.95 | 1.05 | 0.860 | 1.06 | 0.99 | 1.14 | 0.089 | 1.10 | 1.01 | 1.20 | 0.021 |
|  | Single vs. Married | 1.04 | 1.01 | 1.07 | 0.021 | 1.07 | 1.02 | 1.12 | 0.005 | 1.09 | 1.03 | 1.16 | 0.002 |
| Personal cancer history | Yes vs. No | 0.97 | 0.92 | 1.02 | 0.195 | 0.95 | 0.89 | 1.02 | 0.177 | 0.97 | 0.89 | 1.06 | 0.482 |
| Close family member with cancer | Yes vs. No | 1.07 | 1.04 | 1.11 | <0.001 | 1.13 | 1.08 | 1.18 | <0.001 | 1.19 | 1.13 | 1.26 | <0.001 |

Note: *Mean number of symptoms with delayed response adjusted for the remaining factors. Socioeconomic position: GROUP 1. Directors and managers of establishments with 10 or more employees and professionals traditionally associated with university degrees. GROUP 2. Directors and managers of establishments with fewer than 10 employees and professionals traditionally associated with university degrees. GROUP 3. Intermediate occupations: employees of the administrative type and professionals supporting administrative management. GROUP 4+5 (merged due to small sample size). Free-lancers/self-employed. Supervisors and workers in qualified technical occupations. GROUP 6. Qualified workers of the primary sector and other semi-qualified workers. Group 7. Unskilled workers.

Table S6. Relative score increases (RSI) for the effect of wave for women and in different age groups derived from multiple Poisson regression analyses with interaction terms on total delay scores without considering the variable breast changes.

|  |  | **Cut-off 1 week** | | | | **Cut-off 2 weeks** | | | | **Cut-off 3 weeks** | | | |
| --- | --- | --- | --- | --- | --- | --- | --- | --- | --- | --- | --- | --- | --- |
|  |  | RSI | 95% CI | |  | RSI | 95% CI | |  | RSI | 95% CI | |  |
|  |  |  | Lower | Upper | p |  | Lower | Upper | p |  | Lower | Upper | p |
| Sex | Females | 1.13 | 1.09 | 1.17 | <0.001 | 1.12 | 1.06 | 1.18 | <0.001 | 1.06 | 0.99 | 1.13 | 0.099 |
| Age | 18-24 | 1.01 | 0.92 | 1.10 | 0.865 | 0.80 | 0.70 | 0.91 | 0.001 | 0.77 | 0.65 | 0.91 | 0.002 |
|  | 25-34 | 1.08 | 1.01 | 1.15 | 0.025 | 1.09 | 0.99 | 1.19 | 0.072 | 1.06 | 0.95 | 1.19 | 0.284 |
|  | 35-44 | 1.12 | 1.05 | 1.19 | <0.001 | 1.14 | 1.05 | 1.24 | 0.002 | 1.09 | 0.98 | 1.21 | 0.101 |
|  | 45-54 | 1.05 | 0.98 | 1.11 | 0.167 | 1.04 | 0.95 | 1.13 | 0.431 | 0.92 | 0.82 | 1.02 | 0.112 |
|  | 55-64 | 1.20 | 1.11 | 1.28 | <0.001 | 1.19 | 1.07 | 1.32 | 0.001 | 1.06 | 0.93 | 1.21 | 0.375 |
|  | 65+ | 1.17 | 1.10 | 1.25 | <0.001 | 1.03 | 0.93 | 1.13 | 0.583 | 1.02 | 0.90 | 1.14 | 0.775 |

Table S7. Percentage of respondents reporting each barrier as a function of wave and sex.

|  |  | Sex | |
| --- | --- | --- | --- |
|  | Wave | Male | Female |
| I would be embarrassed | Pre | 6.9% | 6.4% |
|  | Post | 7.6% | 10.3% |
| I would be worried about wasting the doctor's time | Pre | 15.4% | 17.6% |
|  | Post | 19.8% | 24.6% |
| I would be worried about what the doctor might find | Pre | 16.2% | 20.4% |
|  | Post | 24.3% | 29.7% |
| I do not have enough time to go to the doctor | Pre | 34.1% | 32.2% |
|  | Post | 31.8% | 37.0% |
| Additional barrier | Pre | 23.3% | 23.8% |
|  | Post | 32.4% | 35.3% |

Figure S1. Mean predicted delay scores (using 1-week cut-off) and barrier scores for males and females, derived from multiple Poisson regression analyses.

Figure S2. Percentage of respondents who report they would seek help within a week of symptom onset as a function of wave and respondent sex (males vs. females).

Figure S3. Mean predicted delay scores (using 1-week cut-off) and barrier scores for different age groups, derived from multiple Poisson regression analyses.
